# Agroecology-based assembly and function of endophytic bacteria in seeds of *Triticum aestivum*

**DOI:** 10.3389/fmicb.2025.1699093

**Published:** 2025-10-22

**Authors:** Jogdande Sai Prasad, Archna Suman, Dharmendra Kumar, Pushpendra Sharma, B. Ramakrishnan, K. Aswini

**Affiliations:** ^1^Department of Microbiology, ICAR-Indian Agricultural Research Institute, New Delhi, India; ^2^Department of CPB & PHT, ICAR- Central Potato Research Institute, Shimla, India

**Keywords:** seed endophytes, plant growth promotion, antagonism, bacterial colonization, Gfp tracking

## Abstract

The seed, a vital plant organ for its continuation, contains microbial endophytes that develop as part of the early plant microbiome and assist growing seedlings in various ways. In this study, bacterial endophytes from seeds of wheat cultivars grown under different agro-ecological conditions were genotypically and functionally analyzed. Despite environmental differences and cultivars adapted to distinct agroclimatic zones, the endophytic bacterial count ranged from 2.79 to 5.19 Log CFU/g. The dominant seed bacteria belonged to the phylum Firmicutes, with diverse members of the genus *Bacillus*. There were core and niche-specific bacteria among the different agroclimatic zones. The seed endophytic bacteria exhibited hydrolytic enzyme activities, mainly amylase, cellulase, and xylanase. The nitrogen fixation capacity ranged from 0.81 to 32.06 nmol ethylene h^−1^ mg^−1^ protein, while phosphate solubilisation ranged from 147 to 440 μg mL^−1^. Some seed endophytes from the North Western Plains Zone (NWPZ) showed strong antagonism toward *Fusarium graminearum* (52%), *Bipolaris sorokiniana* (35.9%), and *Tilletia indica* (43.4%). The green fluorescent protein (GFP)-tagged endophytic bacteria, when reintroduced to wheat seeds, were observed to colonize and migrate within germinating seedlings, Confirm their potential for internal establishment and movement within the host. These seed endophytic bacteria may offer notable benefits by colonizing root tissues during germination, thereby enhancing plant growth and yield.

## 1 Introduction

The plant-associated microbiota plays a crucial role in the functioning of the plant holobiont, influencing plant development, health, and productivity throughout its entire life cycle (Lavanya et al., 2025; Lu et al., 2025). It contributes to various aspects of plant growth, such as seed germination, nutrient supply, resistance to abiotic and biotic stress factors, and the production of bioactive metabolites (Saikkonen et al., 2004; Li et al., 2010). Endophytic microorganisms reside within the plant system without causing disease symptoms, unlike pathogens, or inducing organ morphogenesis, as some symbionts do. As mostly commensals, they contribute to several beneficial adaptive functions that support host plant growth and survival (Jana et al., 2022; Wang and Zhang, 2023). Seed production is one of the most important stages of plant life history. The seeds of gymnosperm or angiosperm plants (the spermatophytes) germinate to produce new plants, and they too harbor endophytes, just like roots, leaves, and other plant organs. The concept that seeds may serve as the sources of endophytes or pathogens was first launched by Bakker and Schippers (1987). By being seed-borne, these endophytes assure their presence in new plants (Truyens et al., 2015). The general assumption earlier was that the emerging seedling is colonized by microorganisms from its surrounding environment by horizontal transmission from the soil, mainly due to plant recruitment through a specific profile of root exudates and its immune system (Berg et al., 2014). The presence of endophytes in seeds may be due to the transfers that may occur through vascular connections among different plant organs or from environmental niches. The reproductive meristems or gametes may also provide endophytes the opportunities for their colonization of embryos and endosperm (Malfanova et al., 2012; Díaz Herrera et al., 2016). Hence, the seed endophytes can be either early colonizers from the vascular system and stigma of mother plants or late colonizers through contact with the environmental niches. Though the presence of endophytes is unequivocally demonstrated in the seeds of many crops, their contributions to plant fitness and health are only beginning to be established (Singh et al., 2022; Ameen et al., 2024; Pandey and Saharan, 2025). Seeds are important as microbial carriers because they are involved in the transmission of both potential beneficial and pathogenic microorganisms from one generation to another (Johnston-Monje et al., 2016; Aswini et al., 2023). Therefore, seed-associated microbial assemblages are ecologically interesting because they both represent an endpoint and a starting point for community assembly of the plant microbiota. While the transmission of microorganisms from seed to seedling is the primary source of inoculum for the plant, relatively few research groups only have investigated the composition of the seed microbiota and its dynamics during germination and emergence (Wang et al., 2022; Wu et al., 2022). Some studies have shown that seed core microbiome is specific for terroir and emergence. In contrast to horizontal transmission, the relative importance of vertical transmission, which is the acquisition of microbial entities from the parent, in the assembly of the plant microbiota has been relatively unexplored (Compant et al., 2019; Afzal et al., 2024; Araujo et al., 2025).

In seeds, the vertical or horizontal transmission may involve different microbial guilds and affect the host plants differently. These endophytes can explicitly contribute to seed germination and growth before other types of microbial associations can establish in the emerging plants (Kumar et al., 2021, 2024). The co-evolutionary processes suggest that the vertically transmitted endophytes, through direct transfer from parents to progenies, may have conserved properties with active involvement in the preservation and germination of seeds (Truyens et al., 2014). Truyens et al. (2015) suggested that the vertical transmission of microbiota ensures the “continuity of partnership” with the host plants. During seed germination, these endophytes can aid in acquiring plant nutrients and synthesizing plant hormones (Ran et al., 2024) and producing metabolites, such as lipopeptides, for antifungal activities (Ameen et al., 2024). Earlier, reported that the seed endophytes *Paenibacillus* and *Pantoea* from wheat had the potential for plant growth promotion and biocontrol activities against *Fusarium graminearum* (Díaz Herrera et al., 2016). The diverse environmental conditions and food habits of people in India support the cultivation of three types of wheat (bread, durum and dicoccum). Wheat is cultivated under different agroecological conditions worldwide. Information on the seed microbiome (microbial assemblages including endophytes) will recognize the genotype-specific members and variations in their compositions and functional capabilities due to the ecological conditions. The utilization of the beneficial seed endophytes can ensure germination and other developmental processes, and the establishment of other microbial associations, ranging from mutualism to pathogenicity in the emerging plants (Hubbard et al., 2012; Ridout et al., 2019). The assemblages of seed endophytes in different wheat cultivars from these ecological conditions are poorly investigated. The genetic relationships among wheat varieties and environments are a constituent factor for yield evaluation of new genotypes and identifying locations as the yield predictors across ecological conditions under the varietal release systems all over the world (Afzal et al., 2019; Ahlawat et al., 2022). The genotype-environment (G × E) interactions for improved adaptation and sustained yield production of wheat across different ecologies necessitates the understanding of microbiome composition and their functions, thereof being an integral part of the holobiont (Gdanetz and Trail, 2017; Trethowan et al., 2018).

Therefore, this study investigated the presence of culturable seed endophytes across various wheat cultivars adapted to six major agro-ecological zones in India. It aimed to taxonomically profile the culturable bacterial endophytes and decipher their functional traits for enhancing plant growth and pathogen resistance. A dataset combining taxonomic and functional information of seed bacterial endophytes from different cultivars was used to identify core beneficial bacteria in wheat seeds. The study also sought to demonstrate the effective colonization of selected wheat seed bacteria in germinating roots using microscopy and to track their transmission to different plant parts using a GFP reporter system. Ultimately, the research aims to validate the beneficial effects of seed-vectored bacteria, which are likely to be naturally recruited by the plant to provide benefits when reintroduced into natural conditions. The study also aimed to demonstrate the effective colonization of selected wheat seed bacteria in the germinating roots of seedlings using microscopy and further transmission in different plant parts using *gfp* reporter system. The study will confirm the beneficial impact of seed-vectored bacteria, which will probably be preferentially recruited by the plant to derive benefits when reinoculated in a natural system.

## 2 Materials and methods

### 2.1 Ecology, wheat genotypes, seed sourcing and processing

Sourcing of wheat seeds for isolation was done from six agro-ecological zones, and 20 cultivars were selected in total from all the zones. Four different locations in each zone that have characteristic features related to the total area and productivity, number of varieties released, and major constraints of wheat cultivation were selected. In India, the zones are classified based on soil characteristics, rainfall patterns, temperature, and terrain as the Northern Hill Zone (NHZ), North-Western Plains Zone (NWPZ), North Eastern Plains Zone (NEPZ), Central Zone (CZ), Peninsular Zone (PZ), and Southern Hill Zone (SHZ) (Table 1). The composite seed samples of each cultivar were pooled, and 15 seeds were randomly chosen for isolating endophytic bacteria. The seeds were surface-sterilized separately using sodium hypochlorite (4%) for 3 min with shaking, followed by washing with sterile distilled water. Then, these seeds were immersed in ethanol (75%) for 4 min, followed by repeated washings with sterile distilled water for complete removal of traces of sterilant. An aliquot of the final rinse (100 μl) was tested for the surface sterilization efficiency by plating on the trypticase soy agar (TSA) and incubating at 28 ± 1 °C for 3 days. The surface-sterilized seeds were immersed in sterile water for 1 h to soften the layers for the enumeration and isolation of endophytes.

**Table 1:** Major wheat cultivation zones with the characteristic features of total area, productivity, number of varieties released and constraints.

**Wheat cultivation zone**	**Area (Million ha)**	**Productivity (t/ha)**	**No. of varieties notified**	**Major varieties**	**Major zonal constraints**	**Number of samples**
Northern Hills Zone-**NHZ** (31° 06' 12” N: 77° 10' 20” E)	0.8	16.6	36	VL 616, HS 240, HPW 251, HS 507, VL 907, VL 892, HPW 349	Low temperature, Moisture stress, Low soil fertility, frost damage	15
North Western Plains Zone- **NWPZ** (28° 36' 50” N: 77° 12' 32” E)	11.6	39.4	80	HD 2009, HD 2285, HD 2329, PBW 343, DBW 17, HD 2967, HD 3086	High fertilizer dose, Decline of water table, intensive tillage, terminal heat stress	18
North Eastern Plains Zone -**NEPZ** (25° 51' 39” N: 85° 46' 56” E)	10.5	25.1	55	UP 262, HUW 234, HD 2967, DBW 14, DBW 39, LBW 38, K 0307, K 1006, HD 2733, NW 5054	High temperature, Moisture stress, Micronutrient deficiencies	12
Central Zone-**CZ** (22° 43' 31” N: 75° 51' 55”E)	5.2	24.1	56	Jairaj, Sujata, Swati, HI 1544, HI 8498, MP 4010, GW 322, GW 366, GW 273	Moisture stress, High temperature	6
Peninsular Zone-**PZ** (20° 04' 59” N: 74° 07' 00” E)	1.6	29.8	57	HD 2189, DWR 162, NI 5439, NIAW 917, MACS 6222	High temperature, Imbalanced fertilizer usage, Light soils, Moisture stress	12
Southern Hill Zone-**SHZ** (11° 22' 12” N: 76° 48' 00” E)	0.1	10.1	9	NP 200, HW 5216, HW 1085, HUW 318, HW 517, HD 2135	Moisture stress, Acidic soils	9

[Data of characteristic features collated from Indian Council of Agricultural Research (ICAR)-Indian Institute of Seed Science, Mau, India; www.seedres.icar.gov.in; the number of seed samples of different predominant cultivars used in the present study is given].

### 2.2 Endophytic bacterial isolation from seeds

The culturable bacterial endophytes were isolated and enumerated using trypticase soy agar (TSA) and nutrient agar (NA) media, and the newly developed Wheat Matrix Medium (WMM) (Prasad et al., 2020) that contained wheat flour (1%), yeast extract (0.1%), sodium chloride (0.05%) and agar (1.8%). WMM media is selective enriched media so it favored the microbes associated with the wheat. Seed coats of swollen surface-sterilized seeds were removed gently, using a sterilized scalpel under aseptic conditions, to get endosperm. Then, the endosperms were pressed with the scalpel mildly and placed on agar plates of different growth media; each plate with five endosperms in triplicate for each cultivar tested, were incubated at 28 ± 1 °C for 4–5 days. The bacterial colonies that appeared in and around endosperms were picked up, streaked, and purified on the respective medium plates. For enumerating endophytic bacteria, the surface-sterilized seed endosperm of each cultivar (one gram) was crushed in a sterile mortar and pestle. The standard serial dilution plating technique was followed for counting the CFUs. The pure bacterial colonies, as the wheat seed endophytic bacteria (WSEB), were maintained on the slants of NA as working cultures at 4 °C, and in the glycerol stocks (30%) at −20 °C for further use as described earlier (Robinson et al., 2016).

### 2.3 Phenotyping and phylotyping of wheat seed endophytic bacteria (WSEB)

The phenotyping of WSEB isolates in terms of color, size, and other colony characteristics such as form, margin, elevation and pigmentation on agar medium, as well as Gram staining and spore staining, was done following Bergey's Manual of Determinative Bacteriology (Holt et al., 1994). All the WSEB isolates were screened for their growth potential at different temperatures (4–40 °C), pH (3 to 10), and salt (5–15%) by spot-inoculation of overnight-grown cultures (10^8^ cells mL^−1^)on respective agar plates. The nutrient agar plates amended with polyethene glycol (PEG 6,000 at 10 and 15%) were spot-inoculated and incubated at 28 °C for 48 h to examine the tolerance to drought-like (water-deficit) stress. Testing of phenotypes of these isolates included the hydrolytic enzymes, plant growth-promoting traits and biocontrol activities. All the tests were performed in triplicate, using standard protocols.

### 2.4 Qualitative evaluation for hydrolases

The WSEB isolates were screened qualitatively for hydrolytic enzymes such as amylase, cellulase, xylanase, protease, pectinase, phytase, esterase and lipase by using suitable substrates. Amylase activity was tested using starch agar plates, and cellulase activity was determined using carboxymethyl cellulose according to the methods of Panda and Sahu (1999); Sahu and Mishra (2021) and Wirth and Ulrich (2002), respectively. The selective xylan-agar plates containing 1% (w/v) birchwood xylan were used for xylanase activity (De Clerck et al., 2004). Activities of pectinase were screened on media containing pectin (1%) as the sole carbon source (Hankin et al., 1971). For phytase screening, the plates with medium containing (glucose- 1.5%, NH_4_NO_3_-0.5%, calcium phytate-0.5%, MgSO_4_·7H_2_O- 0.05%, KCl-0.05%, FeSO_4_·7H_2_O-0.001%, MnSO_4_·4H_2_O-0.001%, and agar- 2.0%), was used; lipase activity was observed on the medium composed of peptone (1%), NaCl (0.5%), CaCl_2_ (0.01%) Tween 20 (1% v/v), and agar–agar (1.5%) with pH 7.4, and esterase activity by replacing Tween 20 with Tween 80 in the above-mentioned medium were screened (Plou et al., 1998).

### 2.5 Quantitative estimation of hydrolase activities

For estimation of amylase activity, the fresh cultures of WSEB isolates (eight from each zone) were randomly selected and inoculated into broth containing soluble starch (1%), peptone (0.5%), (NH_4_)_2_ SO_4_ (0.2%), KH_2_PO_4_ (1%), K_2_HPO_4_ (0.2%), MgCl_2_ (0.001%) at pH 7 and incubated on a shaker at 150 rpm for 3 days at 30 °C. After incubation, the culture was centrifuged at 10,000 rpm for 15 min at 4 °C; the cell-free supernatant was prepared to serve as the enzyme source and the amylase activity was assayed by the method of Bernfeld (1955) with some modifications (Miller, 1959). The cellulase activities of cultures were estimated as described earlier (Demissie et al., 2024); the activities of xylanase by the method of Fasiku et al. (2023). The activities of protease and phytase were assayed by the methods of Tsuchida et al. (1986) and Fiske and Subbarow (1925), respectively. Lipase activity was determined using p-nitrophenol palmitate (pNPP) as a substrate described earlier (Karadzic et al., 2006). Citrus pectin (0.5% (w/v) in 0.1M of pH 7.5 phosphate buffer was used for estimating pectinase activity (Rehman et al., 2012). Esterase activity was determined by using hydrolysis of p-nitrophenyl butyrate (pNPB) as a substrate as described by Karadzic et al. (2006).

### 2.6 Phenotyping of WSEB for plant growth promotion traits

The qualitative screening of WSEB isolates for their plant growth promoting (PGP) attributes *viz*. estimation of nutrients solubilization (phosphorus, potassium) and production of phytohormone (indole-3-acetic acid), siderophores, HCN and ammonia was determined by the standard methods (Pikovskaya, 1948; Schwyn and Neilands, 1987; Bacon and Hinton, 2007; Cappuccino and Welsh, 2018), respectively. The nitrogen-fixing potential of the WSEB isolated was tested using the acetylene reduction assay (ARA) (Hardy et al., 1968). The bacterial isolates were assayed under aerobic and microaerophilic conditions by inoculating in semisolid nitrogen-free Jenson's medium into 30 mL vials and incubating for 7 days at 30 °C. Then the vials were sealed with rubber septa, and the gas phase of each vial was replaced with a gas mixture of nitrogen, air, and acetylene (90:10:10, v/v) and cultures were re-incubated at 30 °C for 24 h. The amount of ethylene produced by acetylene reduction was measured in a gas chromatograph (F11, PerkinElmer, USA), and expressed based on protein, determined by the standard Bradford (1976) method. The P-solubilisation was quantitatively estimated by the method of Mehta and Nautiyal (2001). Indole acetic acid production was estimated according to Duca et al. (2014). All assays were done in triplicate using the representative isolates from each zone.

### 2.7 Antagonistic activities of WSEB against fungal pathogens

Three fungal pathogenic strains [*Fusarium graminearum* (ITCC 3437), *Bipolaris sorokiniana* and *Tilletia indica*] were obtained from the Division of Plant Pathology, Indian Agricultural Research Institute, New Delhi, India. The actively growing fungal strains (1 cm^2^ plug) were inoculated on one side of the PDA plates, the other side of the plates were streaked with bacterial endophytes at equidistant points of the plate, and incubated at 25 °C for 7 days. All the WSEB isolates were assessed for antifungal activities using dual cultures on the PDA plates (Ribeiro et al., 2021). The evaluation was performed in triplicate for each fungus.

### 2.8 Phylotyping using 16S rRNA genes

All the selected WSEB isolates (46) were characterized phylogenetically, using the 16S rRNA gene sequencing. The genomic DNA of these isolates was extracted using Zymo Research (ZR) Bacterial DNA MiniPrep™ extraction kit according to the manufacturer's protocol (The Epigenetic Company). The quality of extracted DNA was examined by running it on agarose gel electrophoresis. The 16S rRNA gene was then amplified with forward pA (27F) and reverse pH (1492R) primers using PCR thermocycler (peqSTAR 96, VWR International GmbH, Vienna). The reaction mixtures contained the master mix (10 μl) containing 10 × Taq buffer, dNTPs (10 mM), MgCl_2_ (25 mM) Taq DNA polymerase (1 U), forward and reverse primers (1.5 μL), and genomic DNA (2 μL) and the PCR grade water (5 μL). The thermal cycler was programmed with initial denaturation at 95 °C for 2 min followed by 35 cycles of denaturation at 95 °C for 50 s, annealing at 53 °C for 45 s and extension at 72 °C for 90 s and then, final extension at 72 °C for 7 min. The PCR products were sequenced by Sanger dideoxy method (AgriGenome, TDI Center, New Delhi) and the obtained sequence data were compared with known sequences in GenBank using NCBI-BLAST. Species identification was done based on the percentage similarity with already available sequences in the data base. Also, the identified, partial 16S rRNA gene sequences were submitted to NCBI GenBank under the assigned accession numbers (MT184815- MT184857) (Woese and Fox, 1977; Hassler et al., 2022).

### 2.9 Colonization of WSEB and their visualization in the emerging plants

The surface-sterilized seeds were inoculated by soaking in the 24 h-old bacterial suspensions for 60 min. These treated seeds were placed on soft agar (0.8%) plates, and kept for incubation in the light:dark (12:12) condition at 20 °C for 5–7 days (seedling stage). Three plates with five seeds each were maintained for all cultures separately along with control plates having seeds without bacterial inoculation. The bright-field microscopy of seedling roots was done for visualizing the live isolates of endophytes using two stains, TTC and H_2_O_2_. Each plate with seedlings was flooded with staining solution containing TTC (1.5 g L^−1^), malic acid (625 mg L^−1^) solution in 0.05 M potassium phosphate buffer (pH 7.0) for 10 h, as described earlier (Bacon and Hinton, 2007; Thomas and Reddy, 2013). After incubation, the roots and leaves were excised from seedlings under aseptic conditions, rinsed with sterile water to remove any external organisms, and examined by the vital bacterial staining technique to check whether the cells turned pink or red. Tissue sections were mounted in sterile water, and the images were captured using a bright-field microscope (Dewinter, India). In another set of seedlings, the bacterial internalization in roots of seedlings (1- to 3-week-old) was stained for 10 h by flooding soft agar plates with potassium phosphate buffer (5 ml, pH 6.9 & 100 mM), containing 3, 30-diaminobenzidine tetrachloride (DAB) (2.5 mM) and 5 purpurogallin units mL^−1^ of horseradish peroxidase. The roots and shoots of seedlings were then excised, placed on a slide containing aniline blue/lactophenol stain (aniline blue dye 0.05 g, phenol crystals 20 g, glycerol 40 mL, lactic acid 20 mL, H_2_O 20 mL) and were examined using the bright field microscopy (White et al., 2019).

### 2.10 Tagging and tracking of seed endophytes using GFP expression vector

The green fluorescent protein (*gfp*) plasmid DNA was isolated from *E. coli* using the alkali method (Bimboim and Doly, 1979). The isolated plasmid DNA was checked for quality and its size on agarose gel (1.2%). The competent cells of selected bacterial endophytes (both Gram-positive and Gram-negative) were prepared using CaCl_2_, and MgCl_2_ (0.1 M); the isolated GFP plasmid DNA (10 μL) was mixed gently with competent cells (200 μl). Transformation events were standardized for the Gram-positive and Gram-negative isolates and the transformation efficiencies were calculated. The transferred colonies were re-streaked on NA-Kan_50_ plates and the colonies were observed under fluorescence (Dewinter, India) and confocal laser scanning microscope (Leica DMIRE2 & DM IRB system) using an excitation laser of 488 nm (Argon laser) and collecting the emission band of 500-550 nm for fluorescence. Additionally, colony PCR was performed to determine the insert of the plasmid vector into the transformed cells using mGFP primers forward (TCAGTGGAGAGGGTGAAGGT) and reverse (GTGGTGGTGGCTAGCTTTGT) using PCR thermocycler (peq STAR 96) (Edwards et al., 1989). The phylotyping of GFP-tagged endophytes was performed using the 16S rRNA gene sequencing was determined by the Sanger Dideoxy method (Agrigenome, India), and identified by BLAST.

The GFP-tagged endophytes were bioassayed for their potential to colonize wheat plants, in soft agar plates and hydroponic conditions under aseptic conditions using the Hoagland solution, as described by Elliott and Lynch (1984). Proper guidelines have been followed for laying out the experiment, and destructive sampling was done as per IARI Phytotron guidelines. The surface-sterilized seeds were treated with GFP-tagged bacterial endophytes (10^6^ cells mL^−1^) for 1 h under aseptic conditions. The observations on seed germination were recorded on 24, 48 and 72 h in the plate assay. For the soft agar plate and the hydroponics-based assays, after 12 days, the fresh roots that grew along the inner wall of the plate and leaves were excised from the seedlings under aseptic conditions. The tissue sections of roots and leaves were prepared by cutting into pieces around 1 cm in length with a sterile razor blade, and these sections were kept on the oil-free glass slides with the addition of sterile double-distilled water droplets to avoid dryness. The prepared slides were later observed by confocal laser scanning microscopy. The transmission light was collected to visualize root structure, particularly GFP fluorescence of cells was viewed, and images were acquired with different objective lenses (i.e., 10X, 40X, and 100X) and reconstructed by Leica Confocal Software (LCS 2.6).

### 2.11 Statistical analysis

The 16S rRNA gene sequences of the WSEB isolates showing >99% sequence similarity were grouped into the same OTU (phylotype). The Shannon index (H), Evenness (J), Simpson's index (D) and Chao-1 were calculated as described earlier (Schloss et al., 2009). Principal coordinate analysis (PCA) was performed for different plant growth-promoting attributes of bacterial isolates using the R software (https://www.r-project.org/). Other statistical calculations were done using MS Excel. Sequence alignment and comparison were performed using the program CLUSTAL-W. One sequence from each group was selected as a representative operational taxonomic unit (OTU). The phylogenetic tree was constructed on the aligned datasets using the neighbor-joining method implemented in the MEGA 6 software (Tamura et al., 2013).

## 3 Results

### 3.1 Abundance of culturable endophytic bacteria in wheat seeds

The mean population densities of culturable, bacterial endophytes ranged from 2.79 ± 0.05 to 5.19 ± 0.06 log CFU g^−1^ d.w. seed, with variations due to the type of medium tested and the agro-ecological zones such as Northern Hill Zone (NHZ), North-Western Plains Zone (NWPZ), North Eastern Plains Zone (NEPZ), Central Zone (CZ), Peninsular Zone (PZ), and Southern Hill Zone (SHZ). The total endophytic bacterial population densities were the least in the CZ with the use of nutrient agar (NA) medium, while the highest was in the NHZ with the use of a newly designed Wheat Matrix Medium (WMM) (Table 2). The zonal influences were apparently lesser on the total population densities when cultured using the Trypticase Soy Agar (TSA) medium. In general, the culturable endophytic bacterial populations were lesser in the seeds sampled from the CZ, PZ, and SHZ than those from the NEPZ, NHZ, and NWPZ. The culturability of bacterial endophytes differed among the media used, and additionally, the abundance of these bacteria was typically higher in the seeds from the NEPZ, followed by the NWPZ and NHZ. Based on morphometric analyses including morphology, size, and pigmentation of colonies, a total of 220 endophytes (43, 40, 38, 28, 34 and 37 from the NWPZ, NEPZ, NHZ, CZ, PZ and SHZ, respectively) were selected for biochemical and phylogenetic investigations.

**Table 2:** Population densities of culturable endophytic bacteria and the diversity indices of phylotypes of predominant culturable isolates.

**Wheat cultivation zone**	**Repertoire of cultures** ^**a**^			**Diversity indices of phylotypes**			
	**Medium used**			**Shannon-H**	**Simpson-D**	**Chao1**	**Evenness J**
	**NA** ^*^	**TSA** ^*^	**WFA** ^*^				
**NHZ**-Northern Hills Zone	4.87 ± 0.07^ab^	3.26 ± 0.05^bc^	5.19 ± 0.06^b^	2.72	0.95	16	0.95
**NWPZ**-North Western Plains Zone	4.74 ± 0.02^b^	3.77 ± 0.04^d^	4.32 ± 0.04^a^	2.66	0.95	15	0.95
**NEPZ**-North Eastern Plains Zone	5.02 ± 0.11^a^	4.25± 0.08^a^	4.40 ± 0.05^a^	2.41	0.94	13	0.94
**CZ**-Central Zone	2.79 ± 0.05^e^	3.12 ± 0.03^c^	3.32 ± 0.05^e^	2.08	0.96	14	0.96
**PZ**-Peninsular Zone	3.73 ± 0.06^c^	3.38 ± 0.05^b^	2.99 ± 0.04^c^	2.74	0.96	16	0.96
**SHZ**-Southern Hill Zone	3.14 ± 0.04^d^	3.26 ± 0.03^bc^	4.06 ± 0.07^d^	2.73	0.96	16	0.96

^a^Colony forming units are given as the mean Log CFU + Standard Error. NA represents “Nutrient Agar,” TSA-“Trypticase Soy Agar,” and WFA-“Wheat Flour-based Agar.”

### 3.2 Phenotyping and phylotyping of wheat seed endophytic bacteria

**Figure 1:**
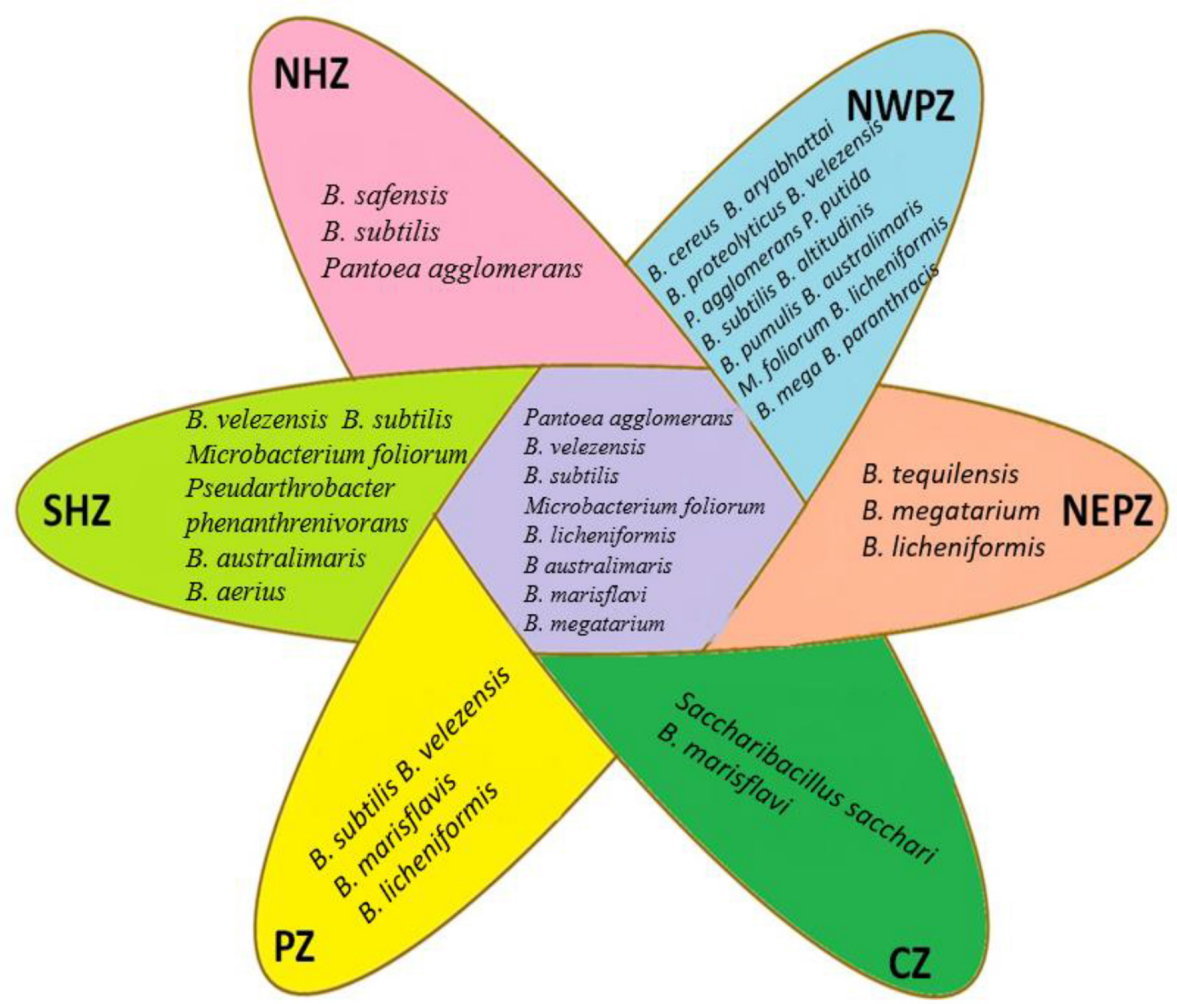
Niche specific and common endophytic bacterial species isolated from different zones.

The phenotyping of Wheat Seed Endophytic Bacteria (WSEB) was performed by examining their growth at different temperatures (4–40 °C), pH (3–10), salt concentrations (5–15%), and polyethylene glycol concentrations (PEG 6000, 10-15%) showed considerable variations in their potentials (Supplementary Figure 1). The most predominant, distinctly different isolates from each zone were further subjected to the phylotyping by sequencing the partial 16S rRNA genes. The accession numbers of the NCBI GenBank for the phylotypes (46) were MT184815–MT184857 (given as Supplementary Figure 2). The diversity indices of these phylotypes in the six major agro-ecological zones showed marginal differences (Table 2). The Shannon diversity (H) value was the highest in the PZ, while those of Chao1 and Simpson's reciprocal indices were the lowest in the NEPZ. The values of species evenness were higher in the NHZ, followed by the NEPZ. Irrespective of these minor differences in the diversity indices, seeds were enriched with three bacterial phyla (i.e., *Firmicutes, Actinobacteria and Proteobacteria* with the distribution ratios of 87.0%, 6.5% and 6.5%, respectively) (Supplementary Figure 3). In the major six cultivation zones, the members of *Bacillus* belonging to the Phylum *Firmicutes* were dominant; other identified species were about three each in *Actinobacteria* and *Proteobacteria*. The niche-specific species were *Bacillus cereus* in the NWPZ, *Saccharibacillus sacchari* in the NEPZ, *B. paranthracis* in the NHZ, *B. australimaris* in the PZ, and B. *aerius* in the SHZ, respectively (Figure 1). The phylogenetic relationships among the identified bacterial species showed that the endophytes were more diverse in the NWPZ than in other zones. Among 16 wheat genotypes used for isolation, the highest 6 isolates were extracted from HD3059 wheat genotypes and out of them, 4 belong to *Bacillus* spp., 1 from *Pseudomonas*, 1 from *Pantoea* spp., followed by 5 isolates from HD3117 wheat genotype and the least number of isolates one in each variety, were extracted from HD2733 and HD3249 wheat genotypes, respectively.

### 3.3 Qualitative and quantitative analyses of phenotypic traits

Culturable isolates of Wheat Seed Endophytic Bacteria (WSEB) exhibited diverse enzymatic activities. Among them, 83% were positive for amylase, 72% for cellulase, 72% for xylanase, 46% for protease, 41% for phytase, 57% for lipase, 67% for pectinase, and 50% for esterase. The endophytic isolates (i.e., NHZ-4, PZ-24, CZ-39, and SHZ-35) were positive for all the lytic enzymes tested. Both NWP-9 and NWP-10 were positive for all the enzymes except lipase, while the isolates such as NEP-19, PZ-27 and NWPZ-60 were positive for all the enzymes except esterase (Supplementary Table 1).

The potentials for hydrolytic enzyme production varied quantitatively among the representative isolates, randomly selected eight each from the wheat cultivation zones (Table 3). The amylase activities of the selected endophytic isolates were between 8.15 and 15.17 nkatal, with the minimum by the isolates from the NEPZ, but the maximum by the isolates from the NWPZ. The highest activities of phytase were by those isolates from the NHZ (5.65 nkatal), but those from the SHZ had the lowest (2.11 nkatal). The pectinase activities were lesser in the representative isolates from the NWPZ (1.01 nkatal), followed by those from the NHZ (2.98 nkatal). The representative WSEB from all other zones had comparable activities. The xylanase activities were between 2.09 and 5.69 nkatal, while the higher activities were in both the NHZ and the PZ while the lower was in the NEPZ and the NWPZ. The cellulase activities were the highest in the PZ (6.64 nkatal), followed by the SHZ (5.86 nkatal) and the NWPZ (5.78 nkatal). The protease activities ranged from 2.78 to 4.79 nkatal, maximum in the PZ, followed by the NHZ. The activities of esterase were more in the culturable bacteria of the NHZ (3.10 nkatal), while those in the NEPZ and CZ had lesser activities (1.20 and 1.88 nkatal, respectively). The lipase activities were the highest in the culturable bacteria from the NHZ (6.55 nkatal), followed by the NWPZ (4.29 nkatal) while those from all other zones had comparable activities (2.34–2.65 nkatal).

**Table 3:** Hydrolytic enzyme activities of representative culturable endophytic bacteria isolated from different wheat cultivation zones.

**Wheat cultivation Zone**	**Amylase**	**Phytase**	**Pectinase**	**Xylanase**	**Cellulase**	**Protease**	**Esterase**	**Lipase**
Norther Hills Zone- **NHZ**	12.61 ± 0.16^c^	5.65 ± 0.13^d^	2.98 ± 0.04^b^	5.69 ± 0.09^d^	3.78 ± 0.06^a^	4.33 ± 0.07^c^	3.10 ± 0.04^c^	6.55± 0.07^c^
North Western Plains Zone- **NWPZ**	15.17 ± 0.16^d^	3.79 ± 0.04^c^	1.01 ± 0.01^a^	2.54 ± 0.03^a^	5.78 ± 0.10^c^	3.30 ± 0.05^ab^	2.54 ± 0.04^b^	4.29 ± 0.06^b^
North Eastern Plains Zone-**NEPZ**	8.15 ± 0.14^a^	3.00 ± 0.04^b^	3.25 ± 0.02^c^	2.09 ± 0.01^a^	4.80 ± 0.10^b^	3.55 ± 0.03^b^	1.20 ± 0.01^a^	2.34 ± 0.03^a^
Central Zone-**CZ**	9.27 ± 0.17^b^	3.72 ± 0.04^c^	3.59 ± 0.05^c^	3.78 ± 0.04^b^	4.69 ± 0.08^b^	2.78 ± 0.05^a^	1.88 ± 0.02^a^	2.61 ± 0.04^a^
Peninsular Zone-**PZ**	13.93 ± 0.24^c^	3.93 ± 0.08^c^	3.26 ± 0.03^c^	5.40 ± 0.05^d^	6.64 ± 0.09^d^	4.79 ± 0.08^c^	2.42 ± 0.04^b^	2.43 ± 0.03^a^
Southern Hill Zone-**SHZ**	8.45 ± 0.09^a^	2.11 ± 0.03^a^	3.97 ± 0.06^c^	4.86 ± 0.04^c^	5.86 ± 0.05^c^	3.03 ± 0.04^a^	4.00 ± 0.07^d^	2.65 ± 0.05^a^

The enzyme activity measurements of the endophytic isolates were expressed in nkatal. The mean values (+ S.E.) followed by the same letter(s) are not significantly different from each other (*p* = 0.05 ANOVA followed by the DMRT test).

### 3.4 Plant growth-promoting traits and antagonism against fungal pathogens

The N_2_ fixing ability and the solubilization of phosphorous and potassium were qualitatively observed in about 46%, 78% and 9% of total culturable bacterial endophytes isolated from wheat seeds, respectively. Likewise, the production of IAA, siderophores, ammonia and HCN production was observed in 89%, 11%, 72% and 59% of the isolates tested, respectively. The NWP-11 isolate possessed all the traits tested qualitatively. The isolate NWP-10 was positive for all traits except ammonia production, while the isolate NEP-22 had an exception of siderophore production (Supplementary Table 2).

The production of indole acetic acid was considerably higher in the culturable bacteria from the NWPZ (203.02 μg mL^−1^) than those from other zones. While the WSEB from the PZ had the least potentials (68.88 μg mL^−1^), those from the NHZ (91.75 μg mL^−1^) and SHZ (111.25 μg mL^−1^), and from the NEPZ (152.25 μg mL^−1^) and CZ (158.36 μg mL^−1^) had comparable potentials for the IAA production. The acetylene reduction activity (ARA), a quantitative estimate of N_2_ fixation of putative endophytes were between 0.81 and 32.06 nmol ethylene h^−1^ mg^−1^ protein (Table 4). The isolates from the NWPZ had the highest potential for the ARA. On the contrary, the endophytic isolates from the NHZ (0.81 nmol ethylene h^−1^ mg^−1^ protein), followed by those from the CZ (4.93 nmol ethylene h^−1^ mg^−1^ protein) were the poorest in their potentials for the ARA. The potentials for the phosphate solubilization were higher in the isolates from the NHZ (415.03 μg mL^−1^) and NWPZ (440.25 μg mL^−1^) than those from other zones. The phosphate solubilization was the least in the WSEB from the SHZ (146.75 μg mL^−1^) (Table 4).

**Table 4:** Plant growth-promoting traits and antagonism against fungal pathogens by the endophytic bacteria isolated from different wheat cultivation zones.

**Wheat Cultivation Zone**	**IAA^1^**	**N_2_ fixation^2^ (ARA)**	**Phosphate solubilization^3^**	**Antagonism against** ^**4**^		
				***F. graminearum***	***B. sorokiniana***	***T. indica***
Norther Hills Zone- **NHZ**	91.75 ± 1.25^b^	0.81 ± 0.02^a^	415.03 ± 5.25^d^	39.71 ± 0.48^c^(7)	41.47 ± 0.37^c^(6)	35.88 ± 0.41^ac^(6)
North Western Plains Zone-**NWPZ**	203.02 ± 3.38^d^	32.06 ± 0.31^e^	440.25 ± 5.63^d^	52.35 ± 0.89^d^(7)	35.88 ± 0.46^ac^ (5)	43.38 ± 0.53^c^(6)
North Eastern Plains Zone-**NEPZ**	152.25 ±1.75^c^	17.94 ± 0.18^d^	203.25 ± 3.38^b^	30.81 ± 0.27^ab^ (6)	29.85 ± 0.38^ab^ (6)	24.63 ± 0.41^ab^ (4)
Central Zone-**CZ**	158.36 ± 1.63^c^	4.92 ± 0.13^b^	219.25 ± 2.01^bc^	26.25 ± 0.31^b^ (6)	34.78 ± 0.54^ac^ (5)	19.12 ± 0.22^b^ (6)
Peninsular Zone-**PZ**	68.88 ± 0.63^a^	20.36 ± 0.23^d^	226.25 ± 1.50^bc^	38.97 ± 0.42^ac^ (6)	29.78 ± 0.46^ab^ (4)	33.75 ± 0.41^ac^ (6)
Southern Hill Zone-**SHZ**	111.25 ± 1.63^b^	9.06 ± 0.06^c^	146.75 ± 2.50^a^	36.54 ± 0.55^ac^ (4)	24.56 ± 0.49^b^ (5)	35.15 ± 0.32^ac^ (3)

^1^IAA-Indole Acetic Acid in μg m^−1^; ^2^ N_2_ fixation (ARA-Acetylene Reduction Assay- nmol ethylene h^−1^ mg^−1^ protein); ^3^P solubilisation μg ml^−1^; ^4^Antagonism in per cent (%). Values in parentheses are the number of isolates that showed antagonism. The mean values (+ S.E.) followed by the same letter(s) are not significantly different from each other (*p* = 0.05 ANOVA followed by the DMRT test).

The endophytes showed variable antagonistic reactions against three potent fungal pathogens tested (*F. graminearum, B. sorokiniana* and *T. indica*). The endophytic isolates from each of these zones, which reacted antagonistically to individual pathogens were tested for percent inhibition. In general, the endophytic isolates from the North Western Plains Zone (NWPZ) had higher levels of percent inhibition against three pathogens tested, followed by those endophytic bacteria from the Northern Hills Zone (NHZ) (Supplementary Table 3).

**Figure 2:**
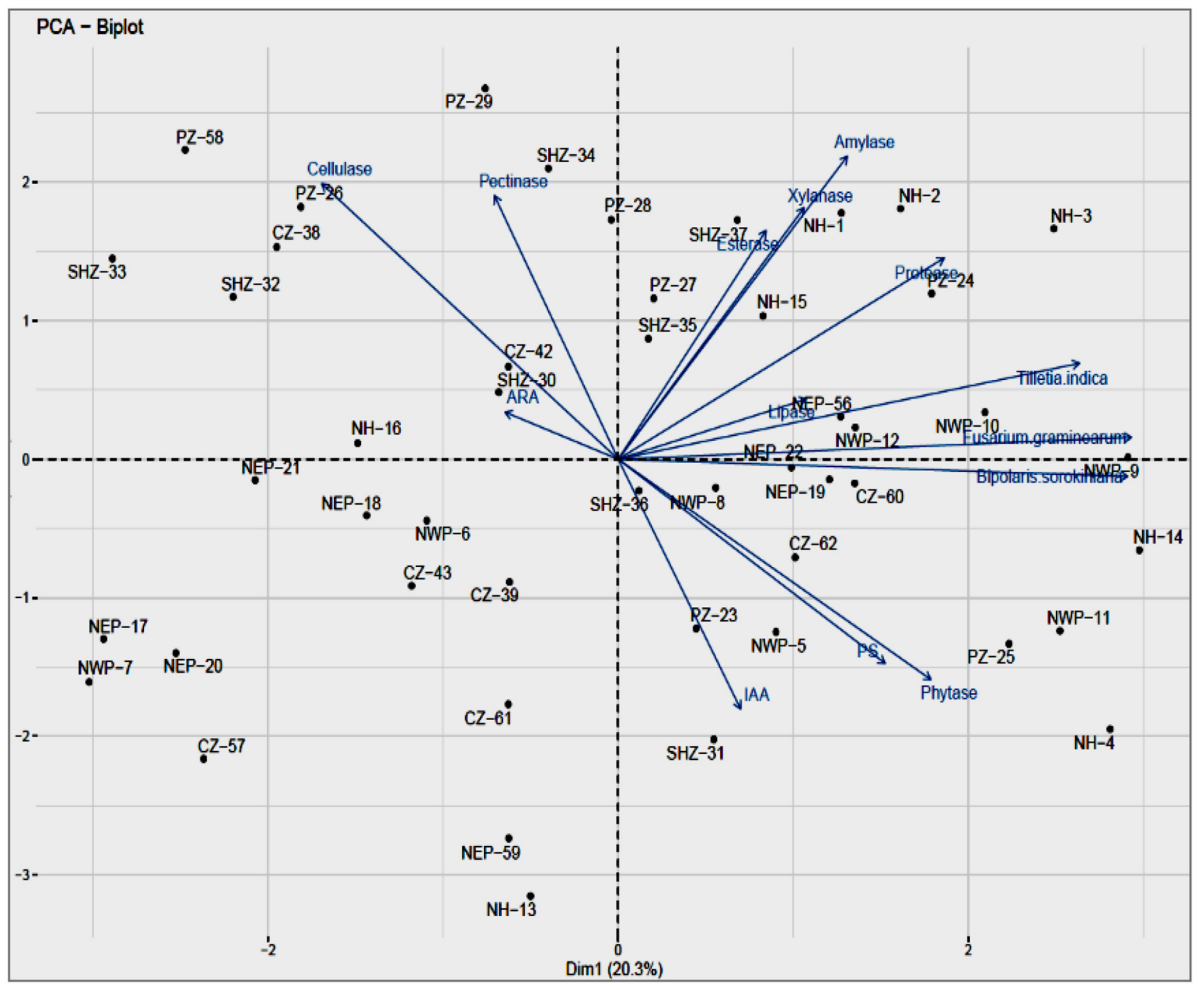
**(a)** Principal coordinate analysis of the different attributes, Biplot showing relationship PGP, lytic enzymes and Biocontrol of bacterial isolates, Component 1 and Component 2 accounted for 22.9% and for 14.5% of the total variation, respectively. **(b)** Variable PCA showing relationship between different activities viz., PGP, lytic enzymes and Biocontrol of bacterial isolates in total variation covered by component 1(22.9%) and component 2 (14.5%).

The principal component analysis was performed for all the phenotypes tested for the representative isolates from the major agro-ecological zones. The analysis showed that the contributions of the first principal component (PC1) were 40.0% while that of the second component (PC2) was 26.25%, together accounting for 66.25% of variations (Figure 2). The principal component (PC1) explained the maximum variation with significant negative correlations in the activities of cellulase and pectinase while those related to the activities of esterase, xylanase, protease, lipase, and phytase, the production of IAA and phosphate solubilization, and the antagonistic activities against *T. indica, F. graminearum*, and *B. sorokiniana* were found positively correlated. The phenotypic trait accounting for PC2 is xylanase, showing maximum variation over cellulase.

The enzyme activities of phytase, lipase and xylanase had a positive correlation whereas amylase was negatively correlated with PC2. The majority of traits (i.e., xylanase, esterase, protease, and lipase activities and antagonism against *B. sorokiniana*) that contributed to the separation of samples had higher coefficients with the NHZ; the activities of esterase and phytase had with the PZ. The representative endophytic bacterial cultures from the NHZ and NWPZ showed maximum variance through the PC1 axis and are considered to be highly efficient with most of the enzyme activities and antagonistic activities against three pathogens tested while those in the NEPZ had less variance, on both sides of the axis, considered to have lesser efficiencies. The analysis also showed that the CZ, SHZ, and NEPZ are closely related and the NWPZ and NHZ as the most diverged from all other cultivation zones.

### 3.5 Colonization abilities of selected culturable isolates of seed endophytes

**Figure 3:**
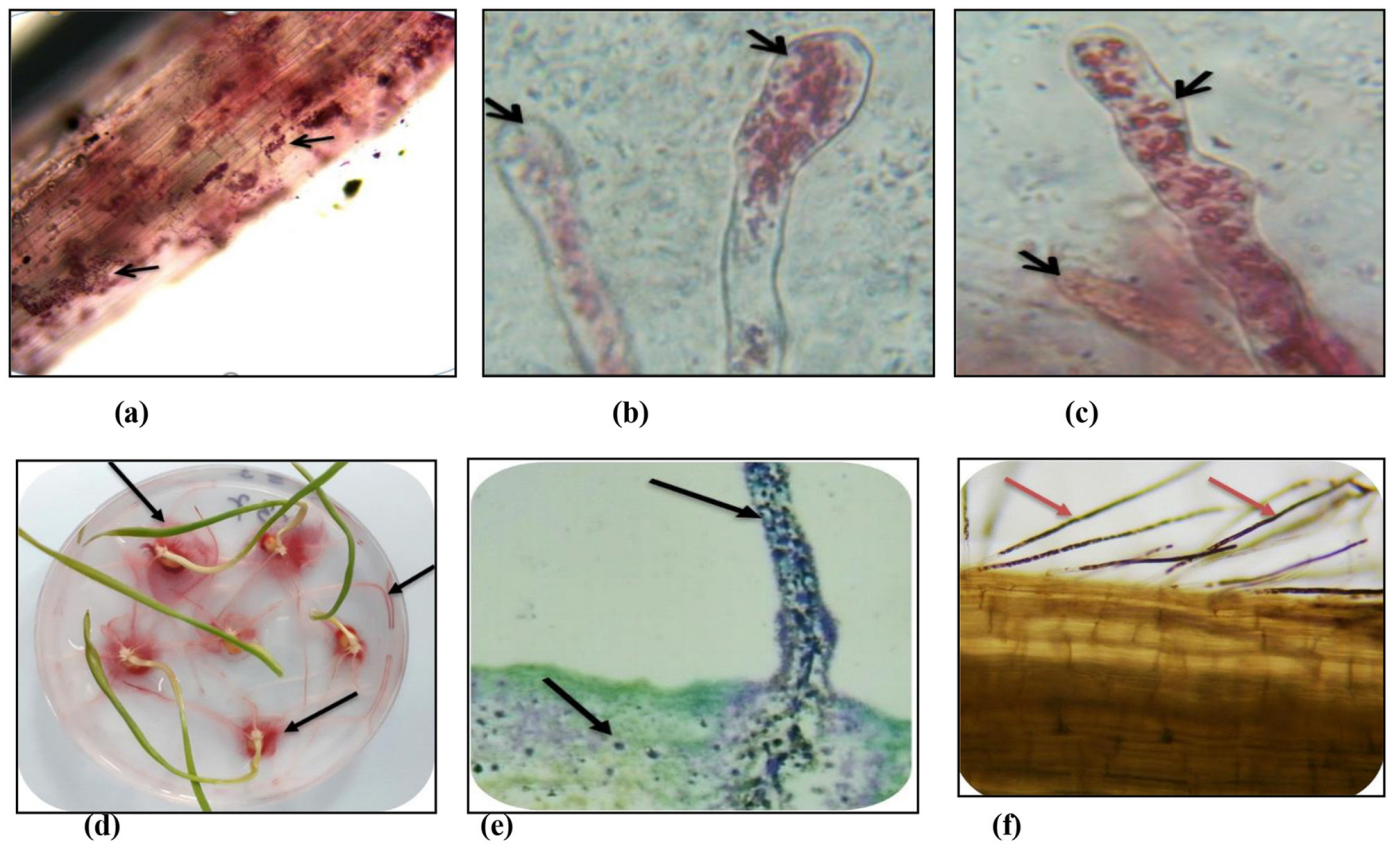
Colonization of bacterial seed endophytes in wheat seedlings evident from TTC and H2O2 treatment. **(a)** main roots (TTC); **(b)** root hair tips (TTC); **(c)** root hairs (TTC); **(d)** seedlings treated with TTC; **(e)** main roots (H_2_O_2_) and **(f)** root hairs (H_2_O_2_).

The root colonization abilities of three endophytic isolates (i.e., *Bacillus megaterium* NEP-22 and one each of *Pantoea agglomerans NWP-9 and Pseudomonas putida* NWP-10), using a non-colonizing bacterial isolate of *E. coli* as the experimental control, were assayed by the TTC staining. The wheat seedlings treated with the selected endophytic isolates showed differential responses as compared to the control and the untreated seedlings. The visual observations after 10 h of treating the seedlings with bacterial cells showed motility, gathering around the root tip first and stained as pink tips of root hairs (Figure 3). Further incubation from 24 to 48 h led to the increased colonization of root hairs and the whole root system. The inoculated bacteria were alive, colonizing the root tips first, and then entering into root hairs. The control treatment using *E. coli* had no pigmentation, neither stained after the TTC or H_2_O_2_ staining. The TTC-stained roots, root hairs, and root sections clearly showed that the bacterial cells of *B. megaterium* NEP-22*, P. agglomerans* NWP-9, and *P. putida* NWP-10 adhered closely, to the surface of epidermal parenchyma, entered intracellularly and subsequently colonized the roots (Figures 3a–c).

The root cells showed the intracellular presence of bacteria, stained dark brown due to the H_2_O_2_ staining, in tissues under the bright field microscopy (Figures 3e, f). The microscopic examinations of seedlings showed the presence of intracellular bacteria in the roots examined. Several bacteria were observed in various locations in seedling tissues, including root hairs, root epidermal cells, and root tips. In all the treated seedling roots, bacteria were seen to be located within cells, located intercellularly in seedling roots and their hairs. Bacteria were observed in root tissues, but could not be visualized in shoot tissues.

### 3.6 Green fluorescent protein (GFP)-tagging of bacterial endophytes in wheat seedlings

**Figure 4:**
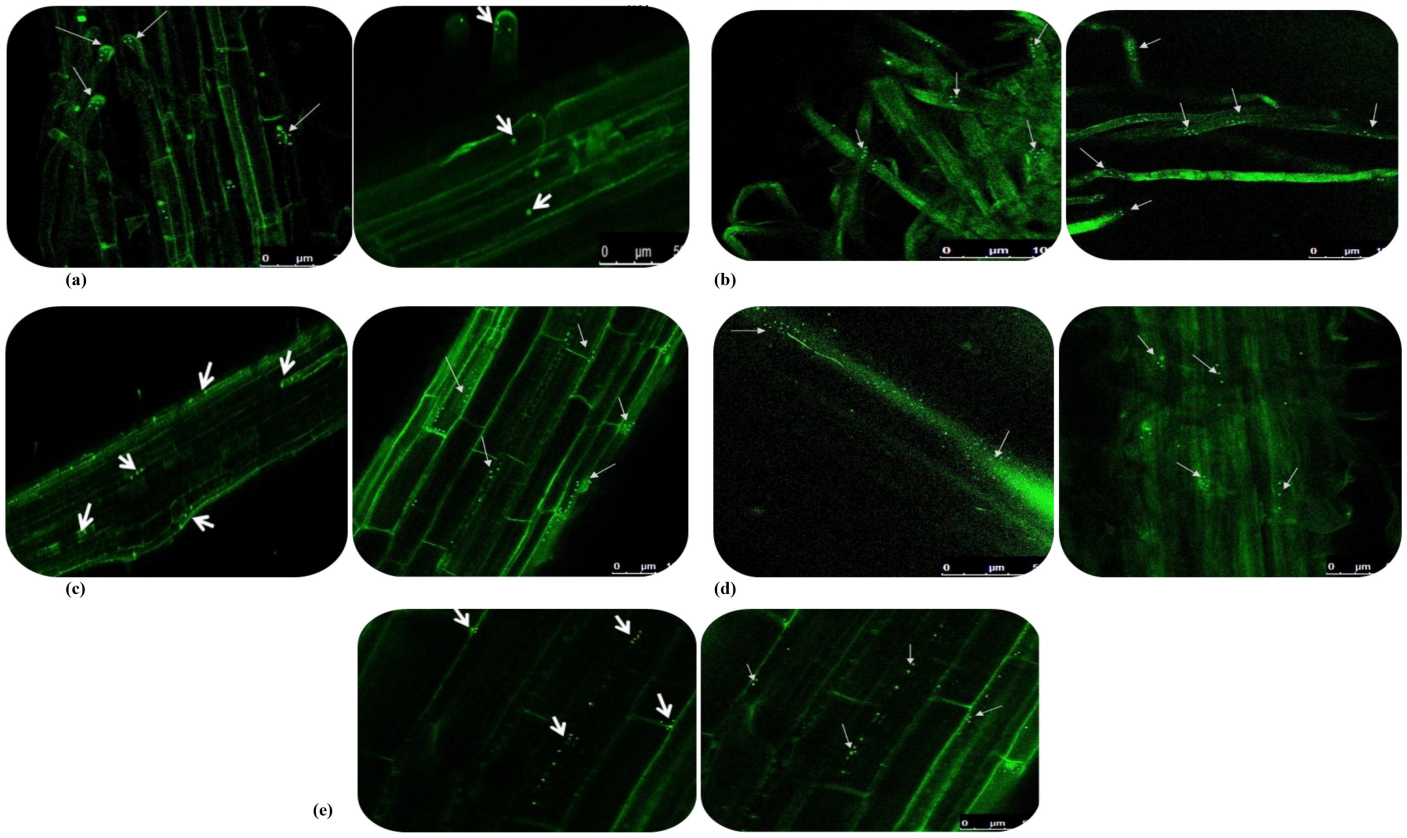
GFP-tagged endophytes tracking in 15-day wheat seedlings under hydroponic condition using confocal microscopy. **(a)** Colonization in root tips; **(b)** Colonization in root hairs; **(c)** Colonization in main roots; **(d)** Colonization in cross sectioning of stem; **(e)** Colonization in leaf of wheat plant.

The five predominant isolates of wheat seed endophytes such as *Bacillus cereus-*NWPZ-5, *Pantoea agglomerans*-NWPZ-9, *Pseudomonas putida*-NWPZ-10, *Bacillus megaterium-*NEP-22, and *Bacillus subtilis-*PZ-23 were selected for the preparation of competent cells and tagged with GFP plasmid vector (obtained from *E. coli* with the GEP plasmid (pCAMBIA1301) of 11 kb) through the transformation process at different temperatures and timings. The bacterial colonies from the Kan_50_ NA plate were prepared for confocal microscopy and the tagged cells were observed as in Figure 4. The colonization patterns of the tagged isolates differed in the tissues. Two of the isolates belonging to the most prevalent bacterial groups (*Pantoea* sp. and *Pseudomonas* sp.) were easier to transform than the other prevalent group of *Bacillus* sp. When the GFP-tagged endophytes were treated with wheat seeds, more light-green autofluorescence was observed in leaves, compared to the root and stem of seedlings. In general, the GFP-tagged cells were more on 7 d after inoculation, than on 5 or 15 d after inoculation. After 15 d of inoculation, the endophytic colonization was more in inter- and intra-cellular spaces in roots, leaves, and the xylem vessels of the stem (Figures 4a–e). The matrix such as the soft agar medium did not affect the colonization pattern while the rapid spread in the vascular system suggested systemic colonization. The seedlings under the hydroponic condition led to greater colonization of GFP-tagged cells in the stem, with lesser in root apices, root hairs, and leaves. But the GFP-tagged bacterial cells colonized the zone of lateral root emergence, root tips, and the intercellular spaces of the root epidermis.

## 4 Discussion

The microbiota associated with wheat crops, especially rhizobacteria and endophytes from different plant organs, have many useful properties such as plant growth promotion, and the mitigation potential for drought, acidity, salinity, or low temperature. The only gammaproteobacterium isolated, identified as a presumptive member of the genus *Pantoea* (Enterobacteriaceae), exhibited notable activity as both an IAA and siderophore producer. In addition, it was capable of phosphate solubilization and was the only isolate able to grow on nitrogen-free medium. Díaz Herrera et al. (2016) and this finding, which was corroborated with our culturable bacterial endophytes in seeds of popular wheat cultivars from the six major agro-ecological zones and showed their beneficial roles and their potential for colonization and growth promotion in germinating plants. The soils of these agro-ecological zones differ in their physicochemical properties (Table 1), and many variations among climatic variables have contributed to the adaptive traits and the popularity of certain wheat cultivars. In the seeds of wheat cultivars (about 21) tested, the composition of culturable bacteria at the levels of genus and species varied, but the common culturable bacterial endophytes suggested that them to be the significant constituent members. In an earlier study by Robinson et al. (2016). The presence of seed-borne microbiota in roots and shoots of the axenically grown seedlings was detected and characterized by isolation and cultivation. Only eight bacterial taxa could be defined at the genus level in their study. On the contrary, a higher diversity of seed bacterial endophytes was observed with the wheat cultivars, popular in each agro-ecological zone in the present study.

In the present study, both the number of endophytic bacterial phyla and the total number of culturable bacteria varied in seeds of different cultivars due to their agroecological factors. In general, the members of the phyla *Firmicutes, Actinobacteria*, and *Proteobacteria* were predominant in the cultivars tested. The most abundant member of *Firmicutes* was the *Bacillus* species (about 40 identified), and other numbers of identified species (three each) belonged to *Actinobacteria* and *Proteobacteria*. The higher abundance of Gram-positive *Bacillus* species may be due to their sporulation ability, while those members of Actinobacteria to the potential for antibiotic production. In addition, wide variations in the G+C content of Bacillus species provide the advantage of better environmental adaptation. Likewise, the members of Proteobacteria are Gram-negative bacteria have diverse metabolic lifestyles ranging from autotrophic to chemolithoautotrophic to heterotrophic. The niches available in seeds are better suited to the members of these phyla. In the present study, only the culturable endophytes were isolated from wheat seeds and characterized. There can be significant differences at the level of the Operational Taxonomic Unit (OUT) in seeds when the yet-to-be cultured members are considered by the sequencing-based methods. In an earlier report on seeds of rice variety (CT6919), the microbiological, physiological, and molecular characterization of about 39 fast-growing seed-borne bacterial flora revealed the diversity of seed-borne mesophiles with the potential for plant probiotic activities such as diazotrophy and antagonism of fungal pathogens (Ruiza et al., 2011). The seed-borne bacteria protect the rice seedlings against the infection of *Curvularia* sp. (Xu et al., 2014) reported that bacteria belonging to several genera showed plant growth promotion (PGP) and biocontrol activities, suggesting the fascinating hypothesis of bacterial–plant co-evolution. The bacterial-plant co-evolution suggests that seed endophytes will have significant roles in the seed health and germination process. The same results of our isolates, such as *Bacillus subtilis* NWP-11 isolate, possessed IAA, siderophores, ammonia and HCN production, N_2_ fixing ability and the solubilization of phosphorus and potassium qualitatively. The isolate *Pseudomonas putida* NWP-10 was positive for all traits except ammonia production, while the isolate *Bacillus megaterium* NEP-22 had an exception of siderophore production. The identification, selection, and utilization of these endophytes can provide better benefits in bacterial inoculation practices.

These endophytic bacteria can directly facilitate the germination process and the proliferation of their plant host through the stimulation and production of phytohormones and hydrolytic enzymes. Indole acetic acid (IAA), the best-characterized auxin among the phytohormones, is essential for the growth and development of plants. In the present study, the seed endophytic bacteria isolated from wheat cultivars from different agro-ecological zones possessed the major functional characteristic of IAA production. The results of the present study corroborated with the report of Khalaf and Raizada (2016) who isolated cucurbit seed-associated endophytes with plant growth-promoting traits such as phytohormone biosynthesis and nutrient acquisition. In addition to phytohormone production, plant growth promotion is mediated by a variety of other mechanisms, including the solubilization of phosphorus, potassium, and zinc; the production of ammonia, siderophores, and HCN (Tilak et al., 2005). There are considerable populations of phosphate (P-) or potassium (K-) solubilizing bacteria observed in seeds of rice, wheat and other crop plants and P-solubilizing bacteria (PSB) can solubilize inorganic phosphate compounds, such as tricalcium phosphate (Vyas et al., 2009). The solubilization of insoluble phosphates in the soil is critical among the nutrient acquisition strategies of crop plants. In the present study, the P-solubilization activity was exhibited by many genera such as *Alcaligenes, Arthrobacter, Achromobacter, Bacillus, Delftia, Methylobacterium, Pseudomonas, Rhodobacter, Staphylococcus*, and *Salmonella*. Most seed endophytes isolated from different wheat cultivars growing under six agroecological zones possessed the P solubilization abilities while relatively a few isolates were also found to be capable of nitrogen fixation in the present study. In another study using different maize cultivars, (Johnston-Monje and Raizada 2011) reported that most of the bacterial isolates from seeds could solubilize phosphorus, secrete acetoin and fix nitrogen. In addition, ACC deaminase activity and antibiosis were found to be moderately conserved among these seed endophytes from different maize cultivars.

The seed endophytic bacteria isolated from different wheat seed genotypes in the present study have multifunctional traits such as hydrolytic enzyme production, plant growth promotion by the synthesis of phytohormones and nutrient acquisition, and biocontrol activity. In another study using maize seeds, Bodhankar et al. (2017) isolated many endophytic bacteria (Maize Seed Endophytic Bacteria, MSEB) from 30 different maize genotypes and found that the dominant genus was *Bacillus* of Phylum Firmicutes, with a few isolates belonging to the genus *Staphylococcus* and an isolate belonging to *Corynebacterium* species of Phylum Actinobacteria. The isolate of *Corynebacterium* species exhibited multifunctional traits related to plant growth promotion and activities such as antagonism against phytopathogenic fungi, production of ammonia, and secretion of lytic enzymes. In addition, some of the MSEB exhibited tolerance to salinity (10%), osmotic stress (40% PEG6000), and temperature (60 °C) (Bodhankar et al., 2017).

The endophytes isolated from wheat seeds had variable antagonism against three potent fungal pathogens tested (*F. graminearum, B. sorokiniana* and *T. indica*) in the present study. The endophytic isolates from wheat cultivars of the NWPZ (North Western Plains Zone) had higher levels of percent inhibition against the three pathogens tested, followed by those endophytic bacteria from the NHZ (Northern Hills Zone). These insights provide advantages in selecting the cultivars adapted to a particular zone for isolating the biocontrol agents and to understand the prevalence and incidence of pathogens in different agro-ecological zones. In the present study, seeds of different wheat genotypes had endophytes with antagonistic activities against *Fusarium, Bipolaris* and *Tilletia*, three of the most important soil-borne pathogens. Our findings corroborated with the report of Van Den Berg et al. (2017) that a large number of seed-associated endophytes (54%), one-third of *in vitro* tested endophytes, showed antagonism against the phytopathogens tested. In another report Díaz Herrera et al. (2016) isolated endophytes from wheat seeds, such as *Paenibacillus* sp., *Pantoea* sp., and *Bacillus* sp., which significantly enhanced plant growth and also showed resistance against *F. graminearum*. Numerous cucurbit seed-associated bacterial endophytes possess extracellular lytic enzyme activities, including cellulase, pectinase, and protease (Khalaf and Raizada, 2016). The presence of endophytes with extracellular lytic enzymes is relevant as several biocontrol agents exert their antagonistic activity through the secretion of lytic enzymes, protecting the host plants either directly or indirectly. The direct mechanisms involve breaking down of essential complex polymers within the pathogen, such as chitin, protein, cellulose, and DNA (Pliego et al., 2011). Even the lysis products (e.g., chitin fragments) can be indirectly employed in plant protection by eliciting host defense responses (Duran-Flores and Heil, 2016). In the present study, what is interesting to know is how abundant these culturable bacteria are, and how diverse the functional characteristics they possess in wheat seeds. The presence of new isolates suggests their likely contributions to seed development and the ensuing germination process and provides the scope for their utilization as microbial inoculants. Many bacterial seed endophytes isolated from wheat seeds can form endospores, and the majority of them belong to the *Bacillus* group resistant to extreme environmental factors. Compant et al. (2011) observed that the seed endophytes could form endospores, thus protecting them from changing conditions inside seeds. These endophytes have other traits such as cell motility and phytase activity to migrate freely inside the plant and enter the seeds before they harden. Future investigations need to be on cell mobility and endosporulation, along with others.

On inoculation of wheat seedlings with endophytic bacterial suspensions, the root colonization was observed with H_2_O_2_ staining, more in the inoculated seedling roots than the uninoculated roots. The potential to colonize the roots upon inoculation provides stronger evidence of their involvement during the germination process. The possibility of some endophytic bacteria that remained on the surface sterilized control seedlings existed; bacteria in the control roots were however present in low numbers. Paungfoo-Lonhienne et al. (2010) showed that the bacterial entry into cells was accompanied by upregulation of plant cell wall-related enzymes such as cellulases, pectinases, xyloglucan endotransglycosidases, cellulose synthases, and expansins. The involvement of host enzymes suggests that plant cells may engage in phagocytosis to acquire bacteria. In addition to the host involvement, bacteria may also produce the cell wall loosening and degrading enzymes to colonize the interior of plant cells. The present study clearly showed the higher potential of seed endophytes to produce hydrolytic enzymes. Like the endophytic bacteria, many symbiotic bacteria enter plant cells using their cell wall-degrading enzymes. In an earlier study, the capacity of *Klebsiella oxytoca* to endophytically colonize wheat plants correlated with its ability to produce pectinases (Kovtunovych et al., 1999). In the present study, the vital staining using 2,3,5-triphenyl tetrazolium chloride (TTC) showed the intra-tissue presence of some live bacteria that are motile and of those non-motile members. Our results are in agreement with those ofand who demonstrated the detection of live endophytic bacteria using vital staining (Bacon and Hinton, 2007; Thomas, 2011).

In the present study, the colonization of wheat seed endophytes was confirmed through the use of both the H_2_O_2_ and the 2,3,5-triphenyl tetrazolium chloride (TTC) staining methods. The vital staining using TTC for detection of live endophytic bacteria showed the presence of both the motile and non-motile members in wheat seedlings, supporting the earlier report of Thomas (2011) in many other plant species. Generally, the colonization routes of endophytic bacteria include their entry through roots, either through passive penetration (via root tip, side root emergence or pathogen entry sites) or active penetration (using cell wall-degrading enzymes such as cellulase and pectinase) (Elbeltagy et al., 2000; James et al., 2002). It is important to know how the seed endophytes compete with other endophytes from the rhizosphere to colonize the germinating plants. Compared to the root endophytic colonizers in our earlier study (Ran et al., 2024; Pandey and Saharan, 2025), the culturable endophytes are fewer in number in seeds. Generally, endophytes colonize the roots predominantly and are often present in low abundance in leaves or seeds, relative to the rhizoplane colonizers (Rodriguez et al., 2009; Adeleke et al., 2021; Chaudhary et al., 2022). From the perspective of bacteria, the colonization of plant tissues internally is advantageous as there is less competition for plant nutrients, relative to those bacteria which colonize the plant (exterior) surfaces (Morales-Cedeño et al., 2021; Liu et al., 2024). The endophytic bacteria within the plant tissues get better protection from abiotic stresses thanthe surface colonizers (Hallmann et al., 1997; Rybakova et al., 2016). More investigations using the sequencing-based methods are needed to detect, identify, and characterize all the culturable and yet-to-be-cultured microbiome members and their functional capabilities.

## 5 Conclusion

The culturable endophytic bacteria from the seeds of different genotypes provide new insights into their composition and the potential for colonization. In addition, our study showed that genotypes play a profound role in their diversity, with variations in functional traits and priority effects on seedling colonization. The diversity analysis showed that the highest values of diversity indices such as Shannon diversity (H), Chao1, Simpson's reciprocal index and Species evenness (J) were generally in the PZ, followed by the NHZ. These endophytes (WSEB) showed variations in the functional traits such as plant growth promotion related to N_2_ fixation, and phosphorous and potassium solubilization. Of eight hydrolytic enzymes tested qualitatively, the maximum number of isolates were positive for amylase (83%) followed by cellulase (72%), and xylanase (72%); the least number of isolates were positive in phytase (41%). Higher potentials for hydrolytic enzyme production suggest the multifarious mechanisms mediated by these seed endophytic bacteria for colonization and antagonism against plant pathogens. In order to make agriculture productive and sustainable, it would be preferable to use interesting seed microorganisms isolated from domesticated seeds and to integrate them into modern cultivars. This could result in the rehabilitation of modern microbiomes to make crop cultures more resistant and resilient in cultural species of interest and the cultivation methods.

## Data Availability

The datasets presented in this study can be found in online repositories. The names of the repository/repositories and accession number(s) can be found in the article/Supplementary material.
