# Supplementary material for: Agroecology-based assembly and function of endophytic bacteria in seeds of Triticum aestivum
Source: Front Microbiol. 2025 Oct 22;16:1699093. doi: 10.3389/fmicb.2025.1699093 (PMC12586097; doi:10.3389/fmicb.2025.1699093)
Supplement: Supplementary file 1 [file Table_1.docx]

**Supplementary** **Table 1: Estimation of hydrolytic enzyme production of bacterial seed endophytes (IU/ml)**

| **Sl.** | **Isolates** | **Amylase** | **Cellulase** | **Xylanase** | **Protease** | **Phytase** | **Lipase** | **Pectinase** | **Esterase** |
| --- | --- | --- | --- | --- | --- | --- | --- | --- | --- |
| 1 | NH-1 | 1.021 | 0.273 | 0.379 | 0.265 | 0.025 | 0.408 | 0.248 | 0.195 |
| 2 | NH-2 | 1.079 | 0.278 | 0.397 | 0.405 | 0.160 | 0.426 | 0.179 | 0.292 |
| 3 | NH-3 | 1.217 | 0.160 | 0.438 | 0.408 | 0.133 | 0.467 | 0.166 | 0.456 |
| 4 | NH-4 | 0.474 | 0.259 | 0.387 | 0.250 | 0.770 | 0.416 | 0.233 | 0.242 |
| 5 | NWP-5 | 0.381 | 0.361 | 0.038 | 0.084 | 0.155 | 0.074 | 0.176 | 0.160 |
| 6 | NWP-6 | 1.069 | 0.384 | 0.027 | 0.097 | 0.105 | 0.069 | 0.080 | 0.042 |
| 7 | NWP-7 | 0.332 | 0.421 | 0.023 | 0.116 | 0.100 | 0.100 | 0.221 | 0.062 |
| 8 | NWP-8 | 1.177 | 0.314 | 0.047 | 0.118 | 0.467 | 0.287 | 0.067 | 0.191 |
| 9 | NWP-9 | 0.879 | 0.573 | 0.467 | 0.363 | 0.425 | 0.567 | 0.310 | 0.518 |
| 10 | NWP-10 | 1.250 | 0.466 | 0.492 | 0.316 | 0.314 | 0.521 | 0.389 | 0.292 |
| 11 | NWP-11 | 1.216 | 0.353 | 0.022 | 0.281 | 0.363 | 0.063 | 0.098 | 0.041 |
| 12 | NWP-12 | 0.977 | 0.344 | 0.374 | 0.295 | 0.100 | 0.378 | 0.056 | 0.050 |
| 13 | NWP-13 | 0.179 | 0.204 | 0.388 | 0.118 | 0.792 | 0.064 | 0.057 | 0.119 |
| 14 | NWP-14 | 0.792 | 0.169 | 0.243 | 0.363 | 0.785 | 0.544 | 0.037 | 0.181 |
| 15 | NWP-15 | 0.961 | 0.194 | 0.219 | 0.122 | 0.022 | 0.403 | 0.207 | 0.192 |
| 16 | NWP-16 | 0.328 | 0.276 | 0.008 | 0.149 | 0.025 | 0.417 | 0.302 | 0.061 |
| 17 | NWP-17 | 0.179 | 0.193 | 0.005 | 0.099 | 0.045 | 0.099 | 0.082 | 0.043 |
| 18 | NWP-18 | 0.363 | 0.249 | 0.027 | 0.173 | 0.240 | 0.091 | 0.048 | 0.053 |
| 19 | NWP-57 | 0.379 | 0.184 | 0.027 | 0.072 | 0.098 | 0.089 | 0.055 | 0.053 |
| 20 | NWP-60 | 0.461 | 0.176 | 0.265 | 0.264 | 0.321 | 0.214 | 0.247 | 0.062 |
| 21 | NWP-61 | 0.422 | 0.284 | 0.238 | 0.152 | 0.219 | 0.154 | 0.204 | 0.195 |
| 22 | NWP-62 | 0.661 | 0.314 | 0.237 | 0.180 | 0.229 | 0.103 | 0.126 | 0.205 |
| 23 | NEP-19 | 0.500 | 0.323 | 0.028 | 0.479 | 0.279 | 0.206 | 0.285 | 0.061 |
| 24 | NEP-20 | 0.610 | 0.360 | 0.038 | 0.109 | 0.045 | 0.303 | 0.092 | 0.072 |
| 25 | NEP-21 | 0.169 | 0.330 | 0.538 | 0.040 | 0.226 | 0.102 | 0.201 | 0.062 |
| 26 | NEP-22 | 0.792 | 0.236 | 0.296 | 0.315 | 0.075 | 0.069 | 0.298 | 0.158 |
| 27 | NEP-56 | 1.139 | 0.212 | 0.277 | 0.371 | 0.422 | 0.099 | 0.246 | 0.057 |
| 28 | NEP-59 | 0.161 | 0.179 | 0.035 | 0.062 | 0.066 | 0.091 | 0.048 | 0.070 |
| 29 | CZ-38 | 0.379 | 0.414 | 0.272 | 0.448 | 0.153 | 0.069 | 0.045 | 0.063 |
| 30 | CZ-39 | 0.909 | 0.332 | 0.318 | 0.439 | 0.288 | 0.267 | 0.145 | 0.130 |
| 31 | PZ-23 | 0.705 | 0.202 | 0.258 | 0.147 | 0.259 | 0.203 | 0.211 | 0.240 |
| 32 | PZ-24 | 0.994 | 0.381 | 0.328 | 0.381 | 0.360 | 0.169 | 0.197 | 0.088 |
| 33 | PZ-25 | 1.099 | 0.406 | 0.366 | 0.090 | 0.132 | 0.091 | 0.073 | 0.155 |
| 34 | PZ-26 | 0.879 | 0.461 | 0.361 | 0.063 | 0.100 | 0.114 | 0.198 | 0.088 |
| 35 | PZ-27 | 0.445 | 0.684 | 0.340 | 0.404 | 0.245 | 0.183 | 0.387 | 0.066 |
| 36 | PZ-28 | 1.061 | 0.317 | 0.349 | 0.327 | 0.281 | 0.069 | 0.023 | 0.089 |
| 37 | PZ-29 | 0.550 | 0.504 | 0.235 | 0.112 | 0.055 | 0.062 | 0.037 | 0.256 |
| 38 | PZ-58 | 0.179 | 0.158 | 0.028 | 0.054 | 0.078 | 0.050 | 0.067 | 0.050 |
| 39 | SHZ-30 | 0.879 | 0.375 | 0.248 | 0.316 | 0.122 | 0.194 | 0.299 | 0.055 |
| 40 | SHZ-31 | 0.369 | 0.490 | 0.239 | 0.296 | 0.104 | 0.155 | 0.275 | 0.397 |
| 41 | SHZ-32 | 0.804 | 0.330 | 0.286 | 0.152 | 0.033 | 0.069 | 0.407 | 0.415 |
| 42 | SHZ-33 | 0.361 | 0.169 | 0.057 | 0.084 | 0.054 | 0.099 | 0.045 | 0.089 |
| 43 | SHZ-34 | 0.150 | 0.180 | 0.515 | 0.207 | 0.235 | 0.378 | 0.190 | 0.081 |
| 44 | SHZ-35 | 0.679 | 0.379 | 0.486 | 0.316 | 0.216 | 0.264 | 0.299 | 0.210 |
| 45 | SHZ-36 | 1.313 | 0.409 | 0.267 | 0.173 | 0.077 | 0.277 | 0.274 | 0.188 |
| 46 | SHZ-37 | 0.304 | 0.301 | 0.326 | 0.139 | 0.364 | 0.268 | 0.287 | 0.087 |
|  | **CD (1%)** | **0.396** | **0.132** | **0.179** | **0.160** | **0.231** | **0.144** | **0.117** | **0.109** |

**Supplementary** **Table 2: Plant Growth promoting activities of bacterial seed endophytes and biocontrol activities against major wheat pathogens**

| **Sl. No.** | **Bacterial isolates** | **ARA activity^@^** | **P^#^** | **IAA^#^** | **K^©^** | **Sider. ^®^** | **NH_3_^®^** | **HCN^®^** |
| --- | --- | --- | --- | --- | --- | --- | --- | --- |
| 1 | *Bacillus safensis* | - | 0.208 | 0.059 | - | - | + | + |
| 2 | *Bacillus subtilis* | - | 0.406 | 0.048 | - | - | + | - |
| 3 | *Bacillus subtilis* | - | 0.280 | 0.050 | - | - | + | + |
| 4 | *Pantoea agglomerans* | - | 1.051 | 0.316 | ++ | - | - | - |
| 5 | *Bacillus cereus* | 96.46 | 0.163 | 0.362 | - | - | + | + |
| 6 | *Bacillus aryabhattai* | 54.86 | 0.362 | 0.148 | - | - | - | - |
| 7 | *Bacillus proteolyticus* | - | 0.264 | 0.132 | - | + | + | - |
| 8 | *Bacillus velezensis* | 2.64 | 0.213 | 0.152 | - | + | - | + |
| 9 | *Pantoea agglomerans* | - | 0.863 | 0.285 | - | - | + | + |
| 10 | *Pseudomonas putida* | 99.03 | 0.789 | 0.187 | + | + | - | + |
| 11 | *Bacillus subtilis* | 3.48 | 0.754 | 0.088 | + | + | + | + |
| 12 | *Bacillus altitudinis* | - | 0.114 | 0.270 | - | - | - | + |
| 13 | *Bacillus megaterium* | - | 0.941 | 0.124 | - | - | + | - |
| 14 | *Bacillus pumulis* | - | 0.163 | 0.030 | - | - | + | + |
| 15 | *Bacillus subtilis* | 6.48 | 0.229 | 0.106 | - | - | + | + |
| 16 | *Bacillus australimaris* | - | 0.042 | -0.001 | - | - | + | - |
| 17 | *Microbacterium foliorum* | - | 0.040 | 0.097 | - | - | - | - |
| 18 | *Bacillus subtilis* | 7.85 | 0.039 | 0.074 | - | - | + | + |
| 19 | *Bacillus megaterium* | - | 0.169 | 0.082 | **-** | **-** | + | + |
| 20 | *Bacillus subtilis* | 4.64 | 0.309 | 0.083 | **-** | **-** | - | + |
| 21 | *Bacillus paranthracis* | - | 0.432 | 0.321 | **-** | **-** | + | + |
| 22 | *Bacillus licheniformis* | - | 0.070 | 0.092 | **-** | **-** | - | + |
| 23 | *Bacillus tequilensis* | - | 0.123 | 0.138 | - | - | + | - |
| 24 | *Bacillus megatarium* | 27.89 | 0.100 | 0.328 | - | - | - | - |
| 25 | *Bacillus megaterium* | 61.83 | 0.294 | 0.068 | - | - | - | - |
| 26 | *Bacillus megaterium* | 45.98 | 0.145 | 0.337 | ++ | - | + | + |
| 27 | *Bacillus licheniformis* | - | 0.143 | 0.112 | **-** | **-** | - | - |
| 28 | *Bacillus megaterium* | - | 0.742 | 0.064 | **-** | **-** | - | - |
| 29 | *Saccharibacillus sacchari* | - | 0.112 | 0.154 | - | + | + | - |
| 30 | *Bacillus marisflavi* | 29.78 | 0.361 | 0.321 | - | - | + | + |
| 31 | *Bacillus subtilis* | 6.84 | 0.681 | 0.136 | - | - | + | + |
| 32 | *Bacillus subtilis* | 5.32 | 0.320 | 0.000 | - | - | - | + |
| 33 | *Bacillus velezensis* | 7.64 | 0.201 | 0.279 | - | - | + | + |
| 34 | *Bacillus marisflavi* | 64.64 | 0.052 | 0.011 | - | - | + | + |
| 35 | *Bacillus subtilis* | 5.46 | 0.369 | 0.016 | - | - | + | + |
| 36 | *Bacillus velezensis* | - | 0.001 | 0.037 | - | - | + | - |
| 37 | *Bacillus licheniformis* | - | 0.002 | 0.051 | - | - | + | + |
| 38 | *Bacillus licheniformis* | 72.94 | 0.184 | 0.021 | **-** | **-** | + | - |
| 39 | *Bacillus velezensis* | - | 0.024 | 0.091 | - | - | + | + |
| 40 | *Bacillus subtilis* | 5.47 | 0.027 | 0.183 | - | - | + | + |
| 41 | *Microbacterium foliorum* | - | 0.012 | 0.070 | - | - | + | + |
| 42 | *Pseudarthrobacter phenanthrenivorans* | 57.56 | 0.023 | 0.118 | - | - | + | - |
| 43 | *Bacillus australimaris* | - | 0.322 | 0.052 | - | - | + | + |
| 44 | *Bacillus aerius* | - | 0.711 | 0.064 | - | - | + | - |
| 45 | *Bacillus velezensis* | 9.45 | 0.025 | 0.196 | - | - | + | - |
| 46 | *Bacillus velezensis* | - | 0.030 | 0.116 | - | - | + | - |
|  | **CD (1%)** |  | **0.309** | **0.233** |  |  |  |  |

**@-** nmol ethylene h−1 mg−1 protein,**#-**mg/ml production, ©- Solubilization, **®-** Production

**Supplementary Table 3: Biocontrol activities of bacterial seed endophytes against major wheat pathogen**

| **Sl. No.** | **Bacterial isolates** | ***Fusarium graminearum* (%)** | ***Bipolaris sorokiniana* (%)** | ***Tilletia indica* (%)** |
| --- | --- | --- | --- | --- |
| 1 | *Bacillus safensis* NH-1 | 38.82 | 60.59 | 56.47 |
| 2 | *Bacillus subtilis* NH-2 | 34.71 | 44.12 | 42.94 |
| 3 | *Bacillus subtilis* NH-3 | 47.65 | 56.47 | 55.88 |
| 4 | *Pantoea agglomerans* NH-4 | 72.35 | 57.65 | - |
| 5 | *Bacillus cereus* NWP-5 | 68.82 | 51.18 | 67.65 |
| 6 | *Bacillus aryabhattai* NWP-6 | 51.76 | - | - |
| 7 | *Bacillus proteolyticus* NWP-7 | - | - | - |
| 8 | *Bacillus velezensis* NWP-8 | 62.35 | - | 65.88 |
| 9 | *Pantoea agglomerans* NWP-9 | 55.29 | 51.18 | 40.59 |
| 10 | *Pseudomonas putida* NWP-10 | 61.76 | 68.82 | 64.71 |
| 11 | *Bacillus subtilis* NWP-11 | 65.88 | 63.53 | 62.35 |
| 12 | *Bacillus altitudinis* NWP-12 | 52.94 | 52.35 | 45.88 |
| 13 | *Bacillus megaterium* NWP-13 | - | - | - |
| 14 | *Bacillus pumulis* NWP-14 | 44.71 | 70.59 | 46.47 |
| 15 | *Bacillus subtilis* NWP-15 | 44.71 | 42.35 | 40.59 |
| 16 | *Bacillus australimaris* NWP-16 | 34.71 | - | 44.71 |
| 17 | *Microbacterium foliorum* NWP-17 | - | 27.06 | - |
| 18 | *Bacillus subtilis* NWP-18 | 22.35 | 23.53 | 33.53 |
| 19 | *Bacillus megaterium* NWP-57 | - | - | - |
| 20 | *Bacillus subtilis* NWP-60 | 60.00 | 62.35 | 52.35 |
| 21 | *Bacillus paranthracis* NWP-61 | - | 65.29 | - |
| 22 | *Bacillus licheniformis* NWP-62 | 46.47 | 65.29 | 49.41 |
| 23 | *Bacillus tequilensis* NEP-19 | 41.18 | 67.65 | 57.06 |
| 24 | *Bacillus megatarium* NEP-20 | - | - | - |
| 25 | *Bacillus megaterium* NEP-21 | 41.76 | - | - |
| 26 | *Bacillus megaterium* NEP-22 | 68.82 | 41.18 | 61.18 |
| 27 | *Bacillus licheniformis* NEP-56 | 40.00 | 40.00 | 45.29 |
| 28 | *Bacillus megaterium* NEP-59 | 32.35 | 39.41 | - |
| 29 | *Bacillus subtilis* PZ-23 | 45.88 | - | 33.53 |
| 30 | *Bacillus subtilis* PZ-24 | 60.59 | 47.06 | 52.35 |
| 31 | *Bacillus velezensis* PZ-25 | 68.24 | 67.06 | 60.59 |
| 32 | *Bacillus marisflavi* PZ-26 | - | - | - |
| 33 | *Bacillus subtilis* PZ-27 | 47.65 | 39.41 | 33.53 |
| 34 | *Bacillus velezensis* PZ-28 | 55.29 | 45.29 | 52.94 |
| 35 | *Bacillus licheniformis* PZ-29 | 34.12 | 39.41 | 37.06 |
| 36 | *Bacillus licheniformis* PZ-58 | - | - | - |
| 37 | *Bacillus velezensis* SHZ-30 | 45.88 | 47.65 | 40.59 |
| 38 | *Bacillus subtilis* SHZ-31 | 63.53 | 52.94 | 57.06 |
| 39 | *Microbacterium foliorum* SHZ-32 | - | - | - |
| 40 | *Pseudarthrobacter phenanthrenivorans* SHZ-33 | - | - | - |
| 41 | *Bacillus australimaris* SHZ-34 | 57.65 | - | 44.12 |
| 42 | *Bacillus aerius* SHZ-35 | 52.94 | - | 65.29 |
| 43 | *Bacillus velezensis* SHZ-36 | 33.53 | 31.18 | 45.29 |
| 44 | *Bacillus velezensis* SHZ-37 | 38.82 | 64.71 | 28.82 |
| 45 | *Saccharibacillus sacchari* CZ-38 | - | - | - |
| 46 | *Bacillus marisflavi* CZ-39 | 42.94 | 42.94 | - |
|  | **CD (1%)** | **31.798** | **34.792** | **34.546** |

**Supplementary Fig.1: Stress tolerance of bacterial isolates at temperature (30,37, 40^0^C), salt (5,10,15% NaCl) & osmotic pressure (5,10,15% PEG)**


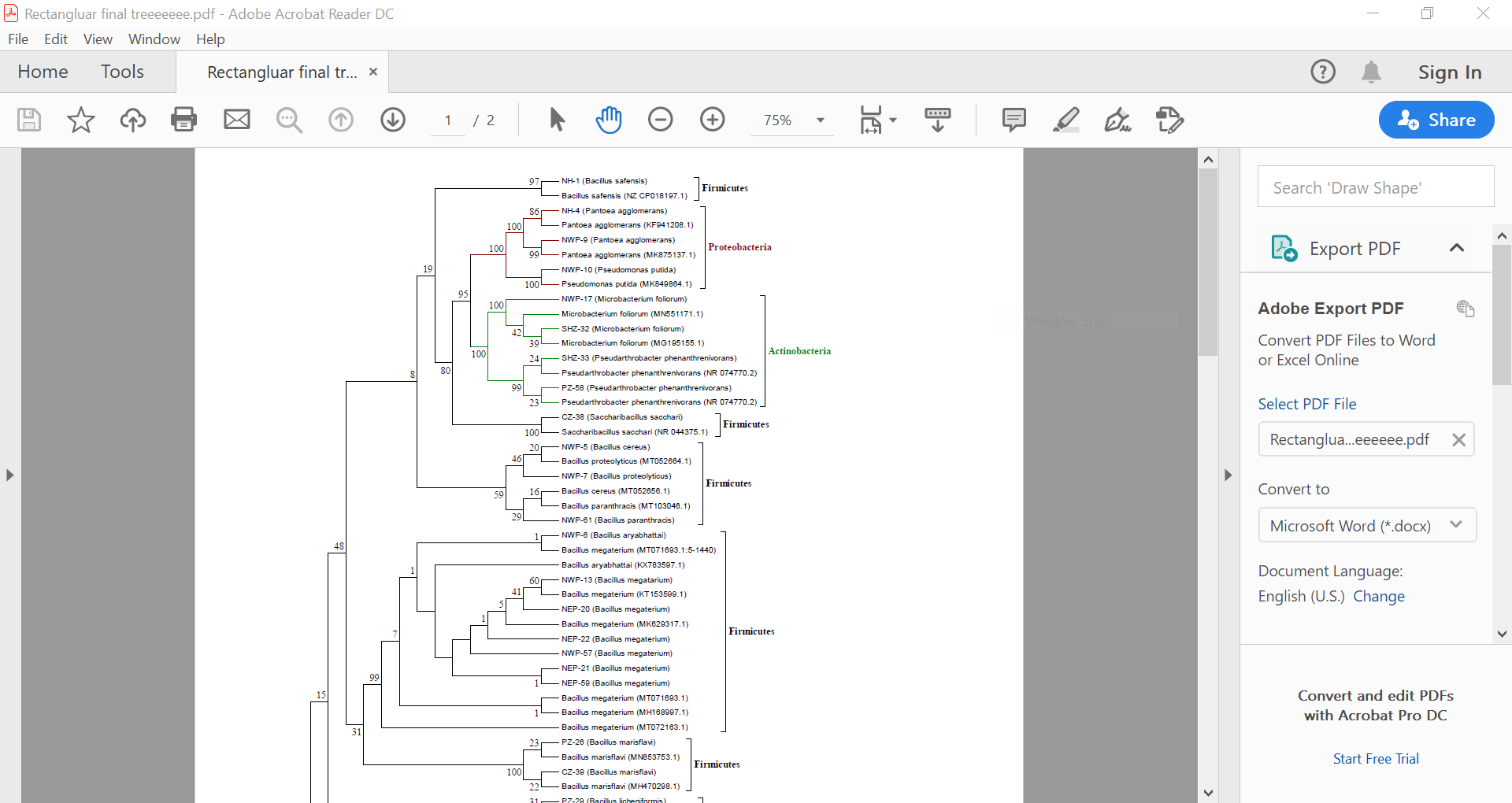


**Supplementary**
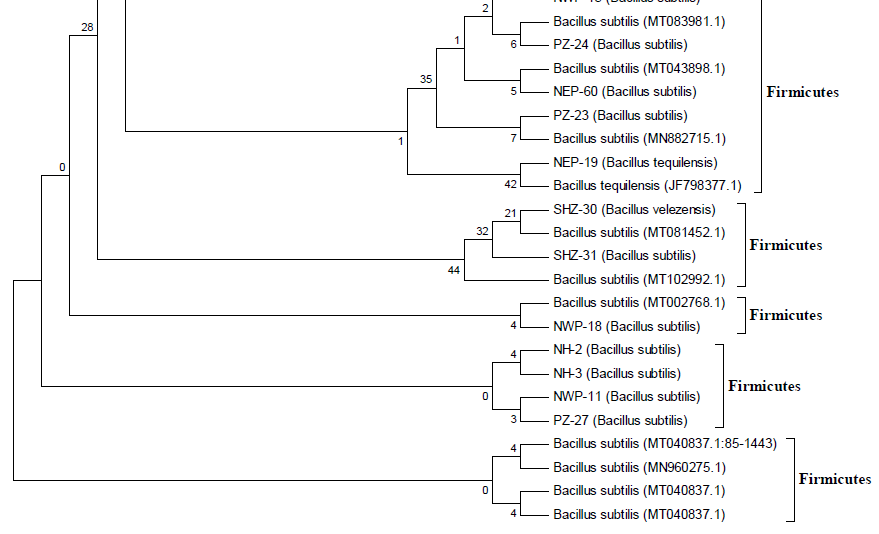

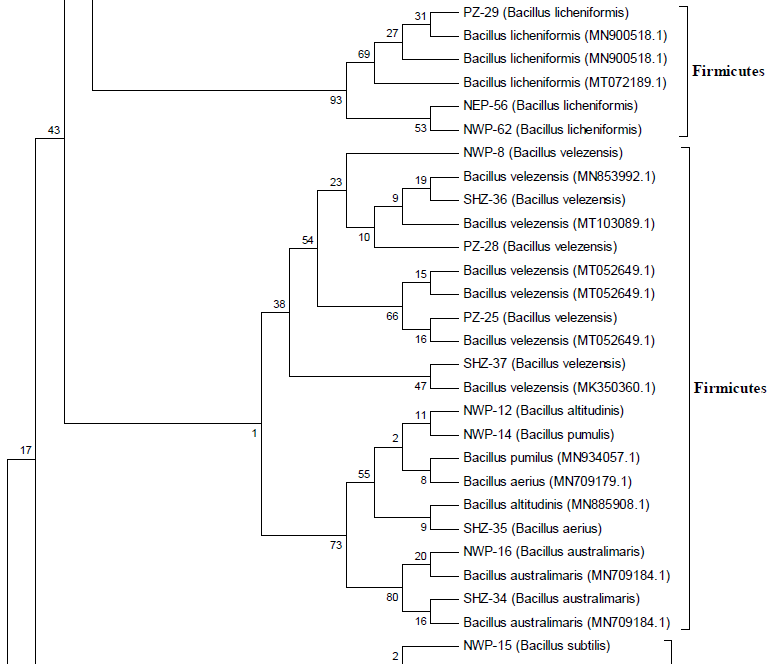
**Fig.2. Phylogenetic tree of 46 bacterial seed endophytes**

Phylogenetic tree showing the relationship among 46 bacteria isolates, 16S rRNA gene sequences with reference sequences obtained through BLAST analysis. The sequence alignment was performed using the CLUSTAL W program and trees were constructed using neighbor joining with algorithm using MEGA6 software

**Supplementary Fig.3: Distribution of phylum and group in the samples surveyed**
